# A prominent crista terminalis associated with atrial septal aneurysm that mimics right atrial mass leading to atrial arrhythmias: a case report

**DOI:** 10.1186/1752-1947-6-403

**Published:** 2012-11-26

**Authors:** Massimo Bolognesi, Diletta Bolognesi

**Affiliations:** 1 Clinical Echocardiography, Internal General Medicine, Asl 112 District of Cesena (Cesena, Italy), Via Ungaretti 494, Cesena 47521, Italy; 2 Via Lambruschini 307, Cesena 47521, Italy

**Keywords:** Atrial arrhythmias, Atrial septal aneurysm, Cardiovascular magnetic resonance, Computed tomography scan, Echocardiography, Prominent crista terminalis

## Abstract

**Introduction:**

The crista terminalis is a variant of normal anatomical structures within the right atrium that mimics an atrial mass on a transthoracic echocardiogram. Atrial septal aneurysm is a rare but well-recognized cardiac abnormality of uncertain clinical significance. The association between crista terminalis and atrial septal aneurysm is unusual but not completely casual. Both anatomical heart structures can lead to atrial arrhythmias.

**Case presentation:**

This case report describes the accidental discovery during an echocardiographic examination of a 64-year-old Caucasian woman who had a left bundle branch block and palpitations.

**Conclusion:**

The clinical relevance of this anatomical evidence in unknown. This was an occasional finding of transthoracic echocardiography, but in this case it is possible to assume its relationship with the occurrence of atrial arrhythmias, and also that computed tomography scan and cardiovascular magnetic resonance is mandatory to define the structure and function of these incidental findings.

## Introduction

The crista terminalis is a fibromuscular vertical ridge of smooth myocardium within the right atrium of the heart [1]. It is located on the posterolateral wall of the chamber. It extends between the right side of the orifice of the superior vena cava inferiorly to the right side of the valve of the inferior vena cava. The echocardiographic finding of a prominent crista terminalis can mimic a right atrial mass, such as a tumor or thrombus [2,3]. Atrial septal aneurysm (ASA) is a rare cardiac abnormality of uncertain clinical significance; it has a variable incidence but transthoracic echocardiographic (TTE) studies estimate the rate to be between 0.08% and 1.2% [4]. It is recognized as a bulging of the thin, billowing septal tissue typically involving the region of the fossa ovalis. ASA is generally considered benign, but it has been associated with atrial septal defects, atrioventricular valvular prolapse [5,6], and atrial arrhythmias. Manifestations attributed to ASA are atrial arrhythmias and arterial embolism. Interatrial septal aneurysm can act as an arrhythmic focus, generating focal atrial tachycardias [4].

Approximately two thirds of focal right atrial tachycardias occurring in the absence of structural heart disease will arise from along the crista terminalis [7]. Descriptions of the association between prominent crista terminalis and ASA are rare [8], and both are important anatomic structures responsible for atrial tachyarrhythmias [9]. In this case report we describe the original association between a prominent terminal ridge, which appeared as a ‘mass’ in the right atrium that needed to be differentiated from a pathological cardiac mass, and a small ASA, in a clinical context of dyspnea and palpitations in a woman with probable atrial tachyarrhythmias. The identification of physiological structures in the right atrium on TTE using subsequent cardiovascular magnetic resonance (CRM) imaging can avoid additional unnecessary, more invasive and expensive tests such as transesophageal echocardiography.

## Case presentation

**Figure 1:**
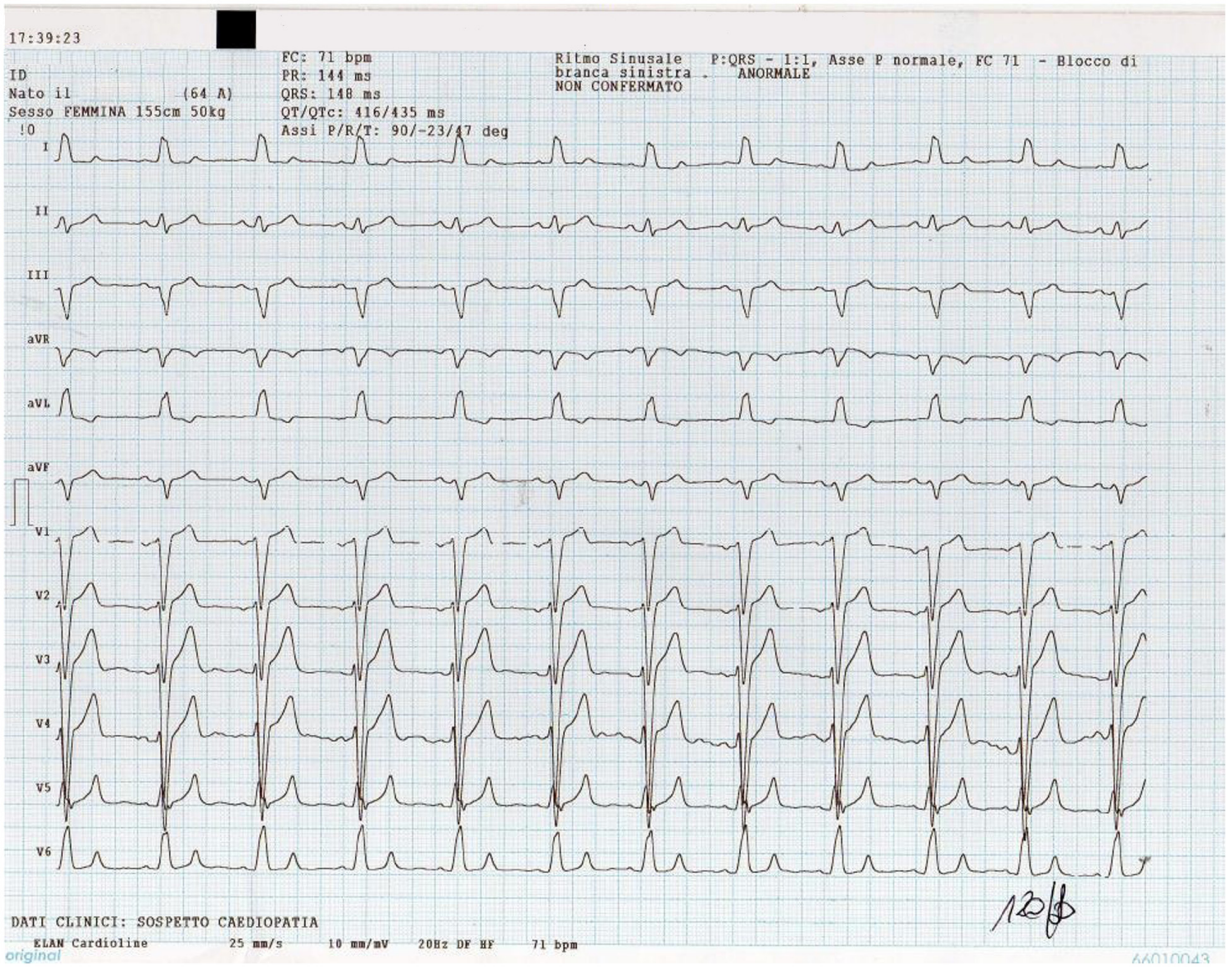
Electrocardiogram track of the patient showing left bundle branch block.

A 64-year-old Caucasian woman, who reported dyspnea and recurrent palpitations, was seen in our office. Her physical examination was unremarkable. Her blood pressure was 130/80mmHg and her pulse was regular at 78 beats per minute. There was no symptom or sign of heart failure and no history of fever or tumor. An electrocardiogram (ECG) showed a left bundle branch block (Figure 1). A chest X-ray showed normal cardiac size and clear lungs. ECG Holter monitoring showed frequent supraventricular ectopic beats.

**Figure 2:**
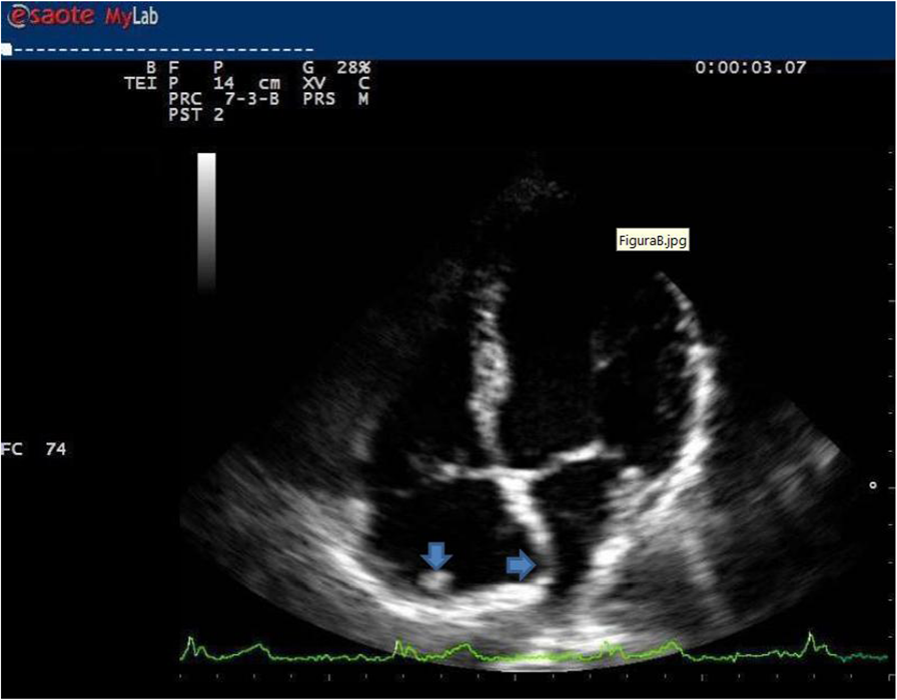
Transthoracic echocardiogram, in four-chamber apical view, shows prominent crista terminalis and atrial septal aneurysm (arrowheads) during atrial diastole.

**Figure 3:**
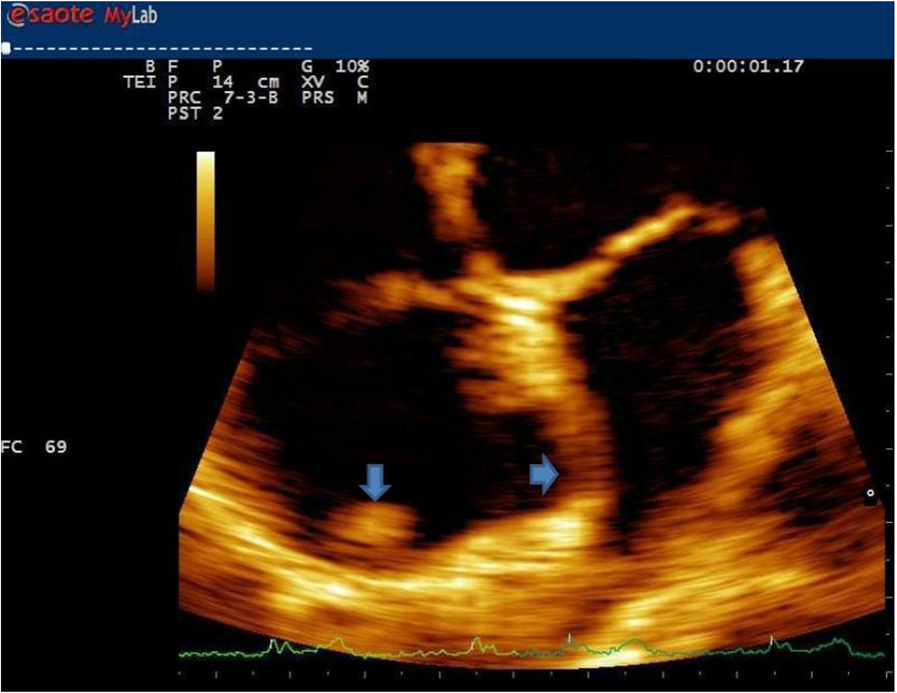
Zoom transthoracic echocardiogram in four-chamber view focusing on crista terminalis and atrial septal aneurysm (arrowheads).

A TTE, in apical four-chamber view, depicts an immobile, round (15mm in diameter) and non-calcified mass on the roof of the patient’s right atrium (Figure 2; zoom Figure 3) mimicking a thrombus or a tumor. A small ASA was also depicted without apparent shunt. The remaining heart structure was normal and showed normal left ventricular systolic function (ejection fraction 62%). Only mild tricuspid regurgitation with a normal pulmonary artery systolic pressure was observed in the absence of septal ventricular dyskinesia. Subsequently a computed tomography (CT) scan was performed.

**Figure 4:**
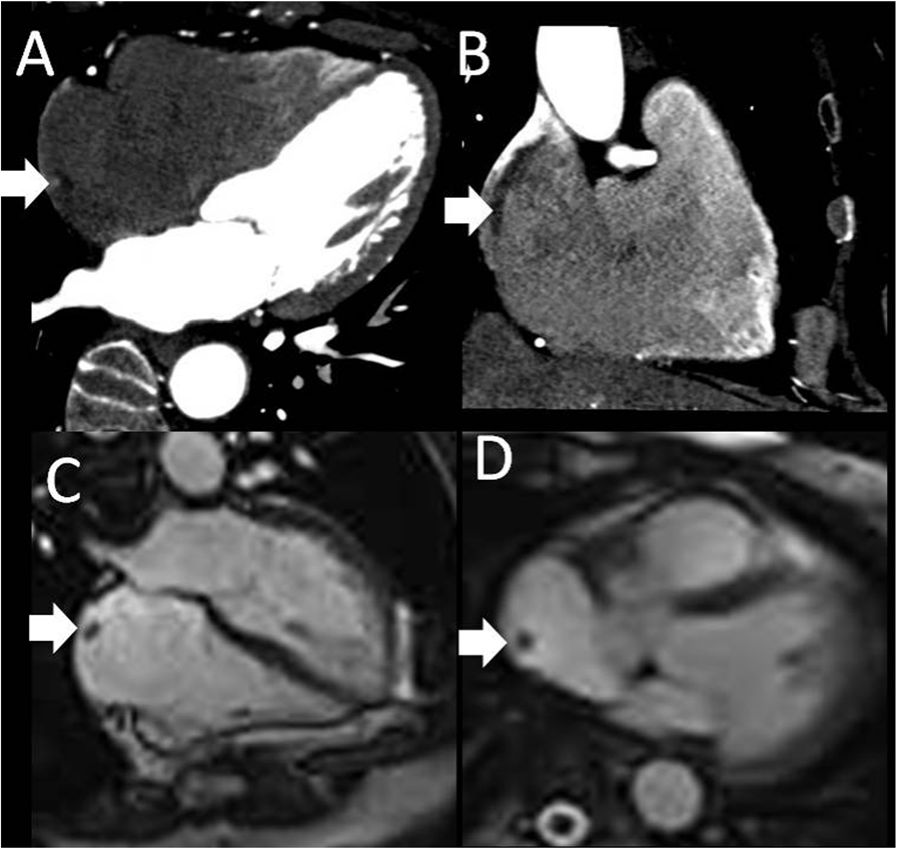
**Computed tomography (A, B) and magnetic resonance (C, D) images of end-diastolic phase showing the prominent crista terminalis (arrows).** In A and B the computed tomography images show the finding in four-chamber view and two-chamber long axis view, respectively. In C and D the steady-state free precession magnetic resonance images show the finding in four-chamber view and axial view, respectively.

CMR imaging was performed (Figure 4). CT and CMR (T1 and T2 sequences) images of end-diastolic phase showed the same findings: a round mass as a prominent ridge localized at the posterolateral region of the right atrium, extending toward tricuspid valve, similar to echo findings and in signal intensity to myocardium; also a small ASA was visualized. An additional computed tomography angiography showed that the patient’s coronary artery was normal.

## Discussion

In a recent report, normal right atrial structures were identified using MRI in 59% of 149 healthy patients. These structures included the Eustachian valve, Thebesian valve, persistent sinus venosus, crista terminalis and the Chiari network [10]. Most of these normal anatomic structures in the right atrium are not visualized on TTE routine standard views. The crista terminalis and ASA are important anatomical structures that can not only mimic pathological atrial mass, but can also be the site of origin of right atrial tachyarrhythmias, referred to as ‘cristal tachycardias’ or paroxysmal atrial fibrillation and atrial flutter, by initiating ectopic atrial beats [7-9]. For these reasons, their association is highly suspected to be the cause of not well-identified arrhythmias, and particularly for the patient described in this report who had a history of recurrent palpitations.

## Conclusion

We consider this report interesting for the following reasons: (1) it concerns a rare anatomical finding (namely the combination between crista terminalis and ASA); (2) the discovery of such an abnormality is incidental; (3) an unusual anatomy can produce atrial arrhythmias; and (4) TTE with subsequently CT scan or CMR are mandatory to define the structure and function of these anatomical findings.

## Consent

Informed consent was collected from the patient for the procedures performed. Consent for data publication was also collected from the patient.

Written informed consent was obtained from the patient for publication of this manuscript and accompanying images. A copy of the written consent is available for review by the Editor-in-Chief of this journal*.*

## Competing interests

The authors declare that they have no competing interests.

## Authors’ contributions

The authors’ contributions were equal in data collection, data analysis, manuscript writing and correction. Both authors read and approved the final manuscript.
